# New dental graduates transition into UK professional practice; a longitudinal study of changes in perceptions and behaviours through the lens of evidence-based dentistry

**DOI:** 10.1186/s12909-024-05182-y

**Published:** 2024-02-26

**Authors:** Waraf Al-Yaseen, Sucharita Nanjappa, Divya Jindal-Snape, Nicola Innes

**Affiliations:** 1 School of Dentistry, College of Biomedical & Life Sciences, Cardiff University, Heath Park, CF14 4XY Cardiff, UK; 2 School of Dentistry, University of Dundee, Park Place, DD1 4HR Dundee, UK; 3 School of Humanities, Social Sciences and Law, University of Dundee, Old Medical School, DD1 4HN Dundee, UK

**Keywords:** Transitions, New dental graduates, Evidence-based practice, University programme, Guidelines, Dentists, Education

## Abstract

**Background:**

This longitudinal study using qualitative methodology aims to investigate the perceptions, and implementation, of evidence-based guidelines into practice among new dental graduates (NDGs) during their transition from university into professional practice, by identifying factors that influence the adoption of evidence-based practice (EBP) in dental practice.

**Methods:**

The study invited NDGs from one UK dental school (*N* = 66) and employed longitudinal, multiple qualitative methodologies for data collection, throughout the participants’ Vocational Dental Training (VDT) year. Initial interviews (Interview 1) conducted upon graduation and follow-up interviews (Interview 2) carried out between six and nine months into professional practice were combined with participants longitudinal audio diaries (LADs) recorded between the interviews. The study.

**Results:**

A total of 12 NDGs agreed to participate. For Interview 1, twelve participants were interviewed, seven of whom agreed to participate in Interview 2 and six recorded the LADs. Interview 1 exposed diverse views among NDGs about EBP, acknowledging its significance but facing obstacles in implementation due to time and financial constraints. They intended to use evidence selectively, often aligning with trainers’ or NHS treatment options, while hesitating to fully embrace EBP in a busy dental practice. During VDT, LAD entries showed initial enthusiasm for EBP, but integrating evidence-based guidelines within the NHS system led to pragmatic treatment decisions, balancing gold-standard and cost-effective options. Over time, NDGs became more comfortable with alternative treatments, considering patients’ financial constraints, yet they expressed frustration with external pressures limiting their clinical decision-making autonomy. In Interview 2, after six to nine months in practice, NDGs exhibited mixed attitudes towards EBP. Some actively used dental guidelines like SDCEP, others associated EBP with hi-tech or expensive materials, while others would thought to rely on colleagues’ recommendations. None consistently sought direct evidence for treatment decisions.

**Conclusion:**

NDGs’ attitudes towards EBP changed and became more negative over their first year in professional practice, leading to challenges in their applying it. It questions the assumption that teaching EBP during undergraduate education ensures its implementation. Further understanding the influences on the development of attitudinal challenges will help to devise effective strategies for fostering lifelong learning and supporting evidence-based practice in dentistry.

**Supplementary Information:**

The online version contains supplementary material available at 10.1186/s12909-024-05182-y.

## **Introduction**

The transition from being an undergraduate student at Dental School to becoming an independent professional dental practitioner is a time of change and can also be a time of stress when trying to establish long-term clinical practice habits [1]. The new graduate needs to adapt to new working conditions, establish relationships with patients and colleagues, and navigate new clinical and administrative processes [2]. This transition can be stressful and challenging, influencing dentists’ decision-making and clinical practice in the long term. Vocational Dental Training (VDT) (the equivalent scheme is called Dental Foundation Training in England and Wales and Northern Ireland) is a supervised training period for newly graduating dentists, serving as a transitional stage from undergraduate education to independent practice. It provides a gradual transition for dental graduates, towards independent clinical decision-making and patient care and completes the shift that generally occurs towards the end of the undergraduate dental programme, towards holistic care rather than single-discipline focussed treatment. VDT is overseen by the UK Committee of Postgraduate Dental Deans and Directors and requires completion of a set of competencies outlined in the *A Curriculum for UK Dental Foundation Programme Training* [3]. The recruitment process in Scotland begins in September when dental students are at the start of the final year of their BDS programme. Interviews for the post take place around May, and the results are usually available in June. The VDT is expected to commence at the start of August. The rest of the UK follows a slightly different timeline, but it is similar in terms of the milestones for new dental graduates on their journey through the application and securing a post.

Evidence-based practice (EBP) is an approach to oral healthcare decision-making that involves integrating scientific evidence, clinical expertise, and patient preferences. Its underpinning principle is to improve health outcome for patients on various levels [4, 5]. Although the evidence for this downstream effect might be weak, it is a fundamental part of lifelong learning.

In the UK, EBP is a fundamental competency expected to be attained upon graduation from dental schools across the UK. Integrated into the General Dental Council (GDC) “Preparing for Practice” standards [6]. The teaching approaches of EBP can vary between dental schools, leading to some variability in how it’s taught and assessed. However, in general, EBP education is introduced early in the dental curriculum starts early and continues throughout the degree, incorporating various educational strategies [7]. Teaching usually covers critical appraisal, literature review, research methodology, and the systematic five-step process for applying evidence in clinical practice. The teaching methods include didactic lectures, interactive problem-based learning, and immersive clinical training.

EBP principles are integrated into clinical experiences to encourage practical application, and students are assessed through written assignments, oral presentations, and Objective Structured Clinical Examinations (OSCEs).

In Dentistry, treatments and materials change as well as the clinical environment, remuneration and patient expectations. Seeking and critically appraising evidence is a crucial aspect of EBP as it supports decision-making to ensure safe and high-quality care. However, the implementation of EBP among dental practitioners varies. with General Dental Practitioners’ (GDPs) perceptions and behaviour towards EBP being mixed, and some evidence of resistance to consulting evidence in practice [8, 9]. 

Several factors influencing GDPs’ adoption of EBP have been identified. Internal elements include a lack of familiarity or confidence in using EBP and time constraints, while external factors include financial resources and practice policies. EBP is a significant component of undergraduate training in UK dental schools, as it is a competence that must be demonstrated to satisfy the requirements of the UK Professional Statutory Regulatory Body, the General Dental Council (GDC) [10]. 

Despite this emphasis on EBP, little is known about the behaviour of new dental graduates (NDGs) related to EBP and how it may change during their transitions to professional practice. Understanding the potential factors that influence the adoption of EBP among NDGs, may provide insights to improve the integration of EBP in dental practice and enhance the quality of patient care. Therefore, this study’s aim was to explore whether NDGs’ behaviour, including their perceptions of, intentions to use, and implementation of, EBP, changed during this transition.

## Method

### Ethics approval and consent

The study protocol was approved by the University of Dundee, Schools of Nursing & Health Sciences and Dentistry Ethics Committee (Application number 2,018,009). Written informed consent was obtained from all study participants.

### Overview of study design

**Fig. 1:**
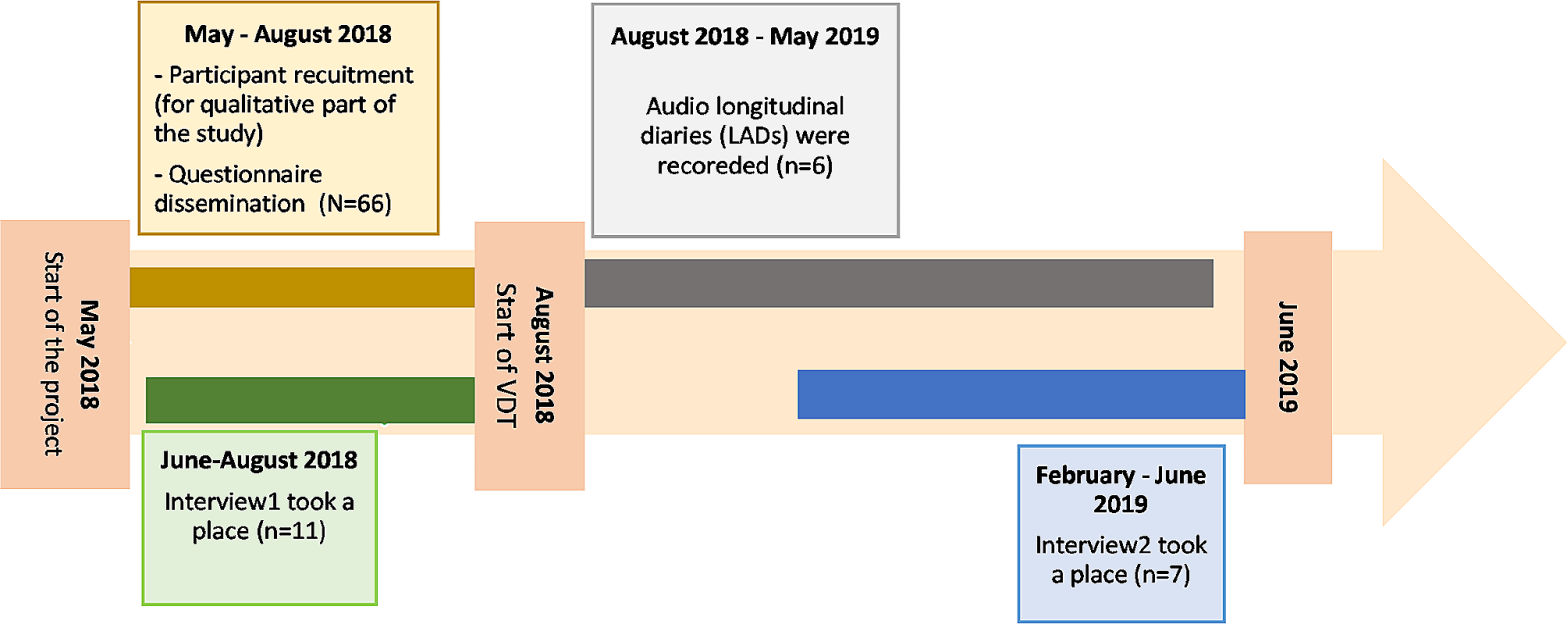
Dental Student Transition to Practice project overview of the three data collection methods. Findings from the questionnaire arm and the qualitative arm related to the exploration of NDGs transition experience has been published [9, 10]

This research was part of a larger programme of research. This research was part of a larger programme of research (See Fig. 1) [11, 12]. A multi-method, longitudinal study was conducted using a qualitative longitudinal methodology, with two data collection methods. Data collection spanned from participants’ graduation until six to nine months into their Vocational Dental Training (VDT) (Foundation Dental Training in England, Wales, and Northern Ireland) [3]. 

Semi-structured interviews were conducted at two time points. The first interview (Interview 1) was conducted shortly after participants had graduated as dentists but before starting their VDT placement. The second interview (Interview 2) took place six to nine months after the NDGs began their 12-month VDT placement.

Longitudinal Audio Diaries (LADs) were recorded by the NDGs during the first six to nine months of their VDT placement.

### Target population and recruitment

The target population for this study was NDGs of the academic year 2018–2019 in the UK. Recruitment took place in one dental school with all NDGs being invited (*N* = 66). Recruitment was initiated by contacting final year dental students who had just completed their final year examinations but had not officially graduated. They were invited to participate via their university email. The invitation email aimed to recruit participants for all three project components and included an invitation letter, a link to a questionnaire asking about evidence-based practice (previously published), a participant information sheet (Appendix 1a), and the consent form (Appendix 1b) for the interviews and LADs. The contact details of the Principal Investigator were included in the email and the NDGs were asked to contact them if they were interested in participating, to schedule a time and location for the interviews.

The recruitment process for Interview 1 was open-ended, starting with the invitation email and continuing with a snowball sampling strategy which involved participants helping to recruit other final year students through their informal and social networks. Snowball sampling was deemed to be the most suitable method due to the unique circumstances of the target sample. Our participants, newly graduated dental professionals entering their Vocational Dental Training, were undergoing a transitional phase characterised by significant life changes, such as relocating to different cities. This posed a challenge when recruiting participants through purposive sampling, which typically relies on predefined criteria. Snowball sampling enabled us to leverage existing connections and networks, making participant recruitment more feasible during a period of geographic mobility and life transition.

No sample size estimation was conducted prior to the study. Recruitment was stopped when no new topics were discussed in the interviews. Recruitment for Interview 2 was conducted by inviting Interview 1 participants to take part in the follow-up interview. Participants for the LAD component of the project was also recruited through the initial invitation email, and recruitment continued through a snowball sampling strategy until the start of the VDT placement.

### Methods of data collection

#### Semi-structured interviews

Before the interview, NDGs reviewed and signed an informed consent form, with the opportunity to ask questions. Participants were offered face-to-face interviews in a quiet and convenient place within the dental school premises or online via Skype. A topic guide was used to outline discussion areas (Appendix 2); participants were asked to freely expand on other topics that were important to them. The interview interactions were conversational in nature and were recorded using a digital audio recorder.

#### Longitudinal audio diaries (LADs)

LADs were also employed to ensure that participants could capture incidents and experiences most relevant to them, contemporaneously. For their convenience and ease of availability, participants were asked to record their LADs using their smartphones and send the recordings to the Principal Investigator through end-to-end encrypted messages via WhatsApp. Written diaries were also offered to those who preferred to submit their data in a written format via email.

Prior to starting recording, there was discussion with the participants about the importance of recording meaningful occurrences to them during their daily practice. Meaningful in this context was defined as any event/interaction, whether at work or outside work, that the participants remembered or that left an impact on their feelings or behaviours. Fortnightly reminders were sent to the participants (solicited entries). However, they were also encouraged to send their reflections whenever they felt they had something to say (unsolicited entries).

### Data handling

The interviews and LADs were transcribed using a non-verbatim approach, focusing on capturing the essential meaning of the spoken words while rectifying grammar errors and omitting non-essential elements. This method ensured accurate and concise transcriptions for efficient data analysis. Recordings and transcripts were stored in a password protected online folder in the dental school’s secure OneDrive cloud. Transcripts were anonymised and any personal details within the transcripts that would have identified the participants were replaced with pseudonyms or omitted if it did not influence the transcript context.

### Data analysis

Thematic analysis [13] was employed for data analysis with the aid of NVivo (Version 11.0, Lumivero, Melbourne, Australia) software [14]. Data from Interview 1, Interview 2 and the LADs were analysed separately to look at patterns at particular time points. Then, findings from each timepoint were compiled to allow understanding around the longitudinal aspects of the participants’ transitions. Data analysis was conducted by one member of the research team. To assure validity of the results, a subset of the analysed transcripts were reviewed by a more experienced researcher in qualitative research.

For the interviews, the analysis process was carried out using an inductive approach using a codebook. An initial coding frame of the main topics that were discussed by the participants was created based on the interview questions (main themes). The analysis process was then carried out inductively using reflective analysis approach to develop emerging subthemes according to participants’ inputs and views.

For the LAD data, analyses were carried out using a reflexive thematic analysis approach where themes and subthemes were developed based on participants’ views by iteratively reviewing each text throughout the data collection and analyses process. Reflections from the LADs from one participant with the largest amount of LAD entries will be presented in the results as a case study.

At the data analysis stage, triangulation of the data from semi-structured interviews and LADs was performed. Within each theme, the findings were combined and presented longitudinally at three different times to illustrate the NDGs’ transition journey: (1) upon graduation (Interview 1); (2) during VDT (LADs); and (3) after spending at least six to nine months in practice (Interview 2).

## Results

Participants: Project participants’ demographic information had been previously reported [12]. Briefly, in Interview 1, eleven participants were interviewed for a total of 759 min. The average interview duration was 69 min, with a median of 61 min and ranged from 38 to 129 min.

In Interview 2, seven NDGs participated, with a total interview time of 487 min. The interviews had an average duration of 70 min, a median of 68 min, and ranged from 47 to 97 min. Four interviews were conducted in person, and three were conducted over Skype.

There were, six NDGs who took part in the LADs, providing a combination of audio diaries (five participants) and a written diary (one participant). Overall, there were 47 entries (42 recorded, five written), with each participant contributing between two and 24 entries. The total duration of the entries was 378 min.

### Main findings

**Fig. 2:**
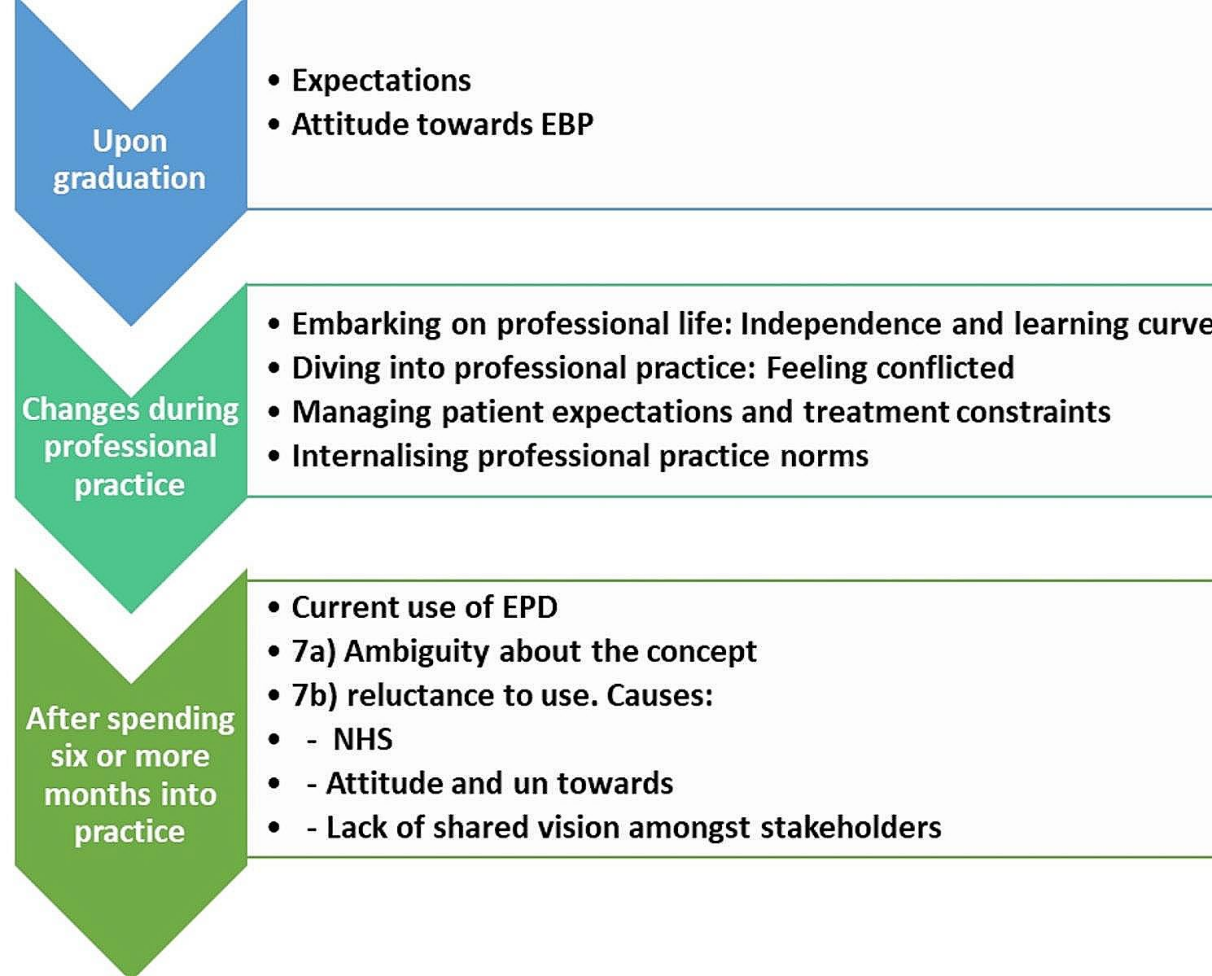
Main themes developed from the collected data (interviews + LADs)

The findings from interviews and LADs are reported chronologically to express its longitudinal nature. The main topic summaries from the interviews are presented. To provide a longitudinal narrative of NDGs’ transitions over time, the LAD data from one participant is presented (See Fig. 2 and Appendix 3).

### Upon graduation and before starting professional practice, findings from interview 1

#### Theme 1- expectations

Participants’ perceptions and expectations to practice in line with available evidence were explored during Interview 1. The opinions were mixed, although there was a general belief that the process of practising in line with the evidence-base would be more challenging in a general practice setting. Participants expected they would consult the evidence during their practice when the circumstances were favourable; mainly if it was in line with the practise philosophy of their trainer or what would be available within the NHS provided treatment.

*I don’t really expect to go by the book. I think I’ll sort of be doing a treatment that would be recognised as common and accepted practice and then proper consent would protect you. NDG 11 -Interview 1*.

**Theme 2: Attitude towards EBP**: Some participants expressed reluctance due to time constraints or their perception of the value of evidence-based practice in a busy general practice setting. They believed that reasonable compromises are acceptable if patients were satisfied with treatment outcomes, regardless of guideline recommendations. Financial constraints were also frequently mentioned.

*I’m starting off VDT, my practice doesn’t do Hall crowns and that’s fair enough. They’re ludicrously expensive… Even though the clinical outcomes for Hall crowns are consistently better. Doesn’t matter, can’t afford them. NDG 6 -Interview 1*.

There was a generally negative perception towards the process of finding and accessing evidence, as it was considered to be complicated and challenging.

*Evidence should be given to you in a way that covers the causes, signs, symptoms, and treatments. It shouldn’t be necessary for me to hunt on Google Scholar or type in weird combinations of words on PubMed. NDG 4- Interview 1*.

### Changes during professional practice, a case study from LADs findings

The diaries of six participants were received with varying lengths and frequencies. From these diaries, the journey of one participant, Tom, will be presented as he provided the most extensive input among the participants, offering deeper insights into his experiences as a new dental graduate starting his professional life. At the time of data collection, Tom was a 23-year-old dental graduate with no previous degree. He provided 41 diary entries totalling approximately 93 min from (August 2018- June 2019).

Theme 3- Embarking on professional life: independence and learning curve: Tom’s initial entries reflected positive and enthusiastic first impressions of his VDT experience. Tom felt a sense of clinical independence and highlighted the opportunity to learn new clinical skills and work with new materials. He also noticed a difference in the involvement of patients in the treatment planning process, which he attributed to the fact that patients paid for their treatments. However, Tom struggled with the NHS payment reimbursement system and found the software difficult to navigate, with complicated treatment coding.

Theme 4- Diving into professional practice: Feeling conflicted: The first two months of Tom’s VDT were busy for him. His LAD submissions were filled with examples of clinical cases and reflections on the changes in his practicing philosophy, and growing awareness of treatment costs. Tom was cautious about certain treatments he considered affordable, like dental pins, which weren’t the recommended best practice. He expressed his anxiousness regarding making these compromises at the beginning and how he aimed to do what is best for his patients.

*I feel anxious about providing these treatments. We’re taught the gold standard, and that’s what I want to provide for my patients. (5 weeks into VDT)*

Tom also found discussing treatment options with patients to be an unpleasant experience. His frustration stemmed from being aware of the gold standard treatment but being unable to offer it under the NHS system. He felt restricted to providing budget-type treatments, which he didn’t appreciate.

*When it comes to providing treatment in practice and knowing what the gold standards are, but then only being able to offer sort of NHS budget type treatment, I really don’t like it. (*6 *weeks into VDT)*

Tom further reflected on this topic two weeks later after encountering situations where he needed to incorporate financial considerations into his treatment planning.

*That’s me done it a couple of times now. It doesn’t get any easier necessarily. You still want to go ahead and do the gold standard, and I think when patients opt for the cheaper of the two options, I feel a sense of pressure to be able to get the same results. (7 weeks into VDT)*

Tom reflected on his new management approaches and shared instances where his trainer suggested alternative treatments that may not be considered best practices.

*A patient comes in with scores of 3 on their BPE (Basic Periodontal Examination)… I knew we should do full periodontal charts and Root Surface Debridement (RSD) and all this. But then my trainer says, ‘What you’re going to do is a deep scrape and polish.’ I struggled with that. It felt like cutting corners because I was fully aware that it wouldn’t effectively address the patient’s gingival condition, and in fact, it might even make it worse. (9 weeks into VDT).*

Theme 5- Patient expectations and treatment constraints: As the demands of professional practice increased, Tom encountered more challenges in his daily practice. He felt frustrated that he needed to refer patients who needed complex treatments rather than treating them himself because he did not have the time to deal with such cases.

*It’s actually a very frustrating attitude, I really don’t like thinking that, ‘Okay this patient isn’t suitable in general practice because I don’t have time for it.*’ *I really would much prefer to have the time to be able to treat all my patients. But I guess that’s just the way it’s got to be at the moment. (10 weeks into VDT)*

Tom was also warned about the legal liability of carrying out less commonplace treatments offered in general practice and was advised to refer such cases to avoid complains.

*…My supervisor says: ‘Well, you can just try it yourself now but when you become an Associate, you might not want to do this and the success is so poor and then if it fails, they’ll blame you’. (8 weeks into VDT)*

Time management was another area of struggle for Tom. He faced challenges with dealing with the NHS payment system that he was not trained to use during his undergraduate training, which added stress to his daily practice as this affected the time available for patient consultations. Tom also believed that he was not fast enough. Tom’s trainer’s inputs about delivering dental education while doing scaling to optimise time management was positively highlighted.

*My trainer has been really helpful, he suggested when you’re doing a scale, you can give oral hygiene instruction. (10 weeks into VDT)*

Similar pieces of advice on time management were evident in other logs.

*My trainer told me that I just need to cut how much I talk at the end of the appointment and trying to wrap things up, because I just generate even more questions. (Tom/11 weeks into VDT)*

Theme 6- Internalising professional practice norms: As weeks passed, Tom gradually aligned his professional identity with the expectations and practices of the general practice. His perspective on treatment decisions became increasingly pragmatic, recognising their higher costs that patients might struggle to afford. This led him to consider alternative options.

*The better treatments are not as cheap for the patient, simply put. Therefore, we offer the pin-retained direct restoration as an alternative, and the patients seem happy with that. (13 weeks into VDT)*

Also…

*We are taught in Dental School that pinned amalgams are the worst thing to do because they can cause fractures. But, in reality, patients don’t want that. They just want you to place a filling and see how many more years they can get out of the tooth without spending too much money. (14 weeks into VDT)*

As the year progressed, Tom became more comfortable with treatment planning that accounted for the financial and time restrains associated with the NHS system and patients’ opinions.

*Dental school taught me how to do gold-standard treatment planning. However, now I need to think more realistically in terms of what is available on the NHS and via the SDR (Statement of Dental Remuneration). Also, I need to consider managing my time in general practice. (16 weeks into VDT)*

Tom’s practice started to settle down with alternative treatments like pin-retained amalgam because it was a popular treatment option for his patients, especially for those with low expectations or budget constraints.

*It works best in situations where you have a patient come in, and they say, ‘I want something done quickly,’ or ‘I don’t want to pay too much,’ or even when they say, ‘Oh, I want to keep it for as long as I can. (20 weeks into VDT)*

He also became more comfortable with referring patients who required more complex treatments.

*I am getting into the groove of regular referrals and knowing where to send them now. (*22 *weeks into VDT)*

After more than six months in VDT, Tom’s practice became a routine. However, he didn’t view this routine positively. He expressed disappointment in how dentists often conform to avoid criticism or patient complaints, and he felt this was due to pressure from external stakeholders.

*It’s a bit disappointing to see how many dentists put themselves in a rut because they all try to do the same as each other to avoid criticism, and they also follow instructions to avoid appearing incompetent. This situation makes me feel like the health board or the GDC has more control over dentists than the dentists themselves. (29 weeks into VDT)*

In one of his final logs, Tom highlighted his frustration with the funding system and costs covered by the NHS system.

*If I were willing to go the extra mile under the NHS, then my trainer and my practice would be losing money because of the amount that we’d get back from the Statement of Dental Remuneration (SDR). It wouldn’t cover my time, and it wouldn’t cover my materials. I just felt really frustrated and really let down by the NHS. (31 weeks into VDT)*

### NDGs behaviour spending six or more months into practice, findings from interview 2

Theme 7-Current use of EPD:

7a) Ambiguity about the concept: Interview 2 presented mixed and varied views regarding the use of EBP among NDGs. While some participants actively implemented EBP in their routine practice, their understanding of EBP differed. Most associated EBP strictly with using dental guidelines, particularly relying on the Scottish Dental Clinical Effectiveness Programme (SDCEP) guidelines. Some participants believed their practice was evidence-based due to using hi-tech equipment and expensive materials that were recommended by colleagues rather than being used on the basis of evidence of their clinical benefits.

*I would say so, for the most part. We’re always trying out new materials or instruments that have been recommended and giving them a go. NDG 1- Interview 2*.

When asked if they tried to look for evidence to inform their treatment decisions, none replied positively. Instead, they relied on alternative sources like peers and VDT trainers, considering them reliable and equivalent to finding and appraising evidence themselves.

7b) reluctance to use: On the other hand, some participants explicitly showed a lack of interest in using EBP or staying updated with the latest evidence. Although they attributed this to a number of reasons, the NHS system was, by far, the main topic that was discussed and blamed for them not keeping their practice focussed on an evidence-based approach. The NHS system was perceived as unfair to both dentists and patients with all participants expressing feelings of stress over their perceived ethical dilemma of providing NHS treatment versus meets the standards of care.

*The NHS is unfairly telling us, ‘Well you’ve got to provide the one that’s the better one. But we’re not going to pay you anything more for it’. NDG 6- Interview 2*.

For patients, EBP was seen as a treatment option exclusive to private dentistry, creating a moral dilemma as patients made economic choices compromising their healthcare.

*It’s creating a moral hazard by steering people in that direction because patients will make economic choices about their healthcare, and they’ll say, ‘Well, the best treatment for me would cost £60, but I don’t really have £60, so I’m going to have to go make a compromise of a treatment for £10’. NDG 4-Interview 2*.

Another limitation highlighted regarding the NHS payment system was bureaucracy. Participants found navigating through the system and process of obtaining approvals for treatment planning as time-consuming and difficult.

*If a treatment plan went over a certain amount of money, then I had to request prior approval before I could continue the patient’s treatment plan. That generally can take quite a long time, and sometimes is quite difficult. NDG 7-Interview 2*.

Another reason for reluctance in adopting EBP, was the perceived disconnect between high standards of care and the business aspects of dentistry. EBP was also often highlighted as a treatment option to be exclusively offered within the private dentistry realm.

*Making speed is a way of compensating for the fact that the standards are poor. You either have high standards or high turnover. That is how you make a business work. NDG 4-Interview*.

Also, guidelines were criticised for being repetitive and lengthy. Others questioned the concept of EBP itself, finding academic papers time-consuming without significant value. Many participants emphasised their professional identity as clinicians rather than academics, explaining their reluctance to engage in the steps of the EBP process.

No, definitely no, the others as well. This is not our job. NDG 3-Interview 2.

A lack of shared vision among stakeholders was highlighted by participants as leading them to feel conflicted between what they could offer and what they perceived as gold-standard treatment.

*I think that each party is just not paying attention to the others. The GDC wants to ensure that everything is happening in a way that protects the patients. Dental schools, on the other hand, focus on teaching us gold standard treatments. But once we start practicing, we have to navigate the realities of working within the NHS and truly understand what we can and cannot accomplish. NDG 1-Interview 2*.

## Discussion

This study provides valuable insights into the changes in perception and adoption of EBP among newly graduated dental professionals, as well as identifying the factors that influence their adoption of evidence-based practices. These insights may help inform strategies to improve the adoption of evidence-based dentistry through teaching methods in dental school curricula and in dental practice.

### Study design and data collection

Our study employed a longitudinal design, gathering data at various time points. The initial interview occurred upon graduation when participants had completed their degree and obtained professional registration with the GDC. This allowed us to explore their perceptions of EBP without the influence of the general practice environment. The subsequent interviews and diary entries were conducted after the participants had spent six to nine months in VDT roles within NHS dental practices, offering insights into the evolution of their attitudes and behaviours toward EBP in their new professional roles.

Two data collection methods, semi-structured interviews and LADs, were employed to ensure a comprehensive understanding of the phenomenon. While the interviews provided focused exploration and opportunities for participants to share their experiences, they generated a substantial amount of complex data. The LADs, though initially challenging to maintain which was reported in similar studies, [15, 16] became a valuable tool for participants to express their feelings and reflect on their experiences, particularly during challenging periods. Some participants also reported a moderate therapeutic effect of recording diaries, similar to findings from other studies using longitudinal diaries [17]. The diaries offered a safe space for reflection on negative experiences and self-doubts. However, the therapeutic effect of LADs can be a limiting factor as it shifts the purpose of diaries from being solely an investigative method to serving as an intervention, potentially influencing participants’ experiences and behaviours [18]. 

Both methods are qualitative in nature, introducing a degree of subjectivity into the data [19]. We took measures to ensure the quality of analysis, including consulting the literature, expert oversight, and internal coding checks. We employed a codebook and reflexive thematic analysis, recognising that the findings are primarily interpreted through the researcher’s lens but remained open to alternative interpretations.

### Main findings

The main findings of this study provide valuable insights into the attitudes and behaviours of dental graduates towards EBP, while also highlighting the challenges they encounter in implementing it. The results reveal a complex and multifaceted picture regarding the incorporation of EBP into the professional work of dental graduates.

During the initial interview (Interview 1), it became evident that dental graduates were not well-prepared to integrate evidence-based practices into their work within the general practice environment. The primary obstacle was the new graduates’ expectation that the workplace environment would differ significantly from dental school in terms of policies, time constraints, and available resources. Additionally, an attitudinal barrier surfaced, as many participants expressed a lack of belief in the role of evidence-based practice in delivering high-quality care. They perceived the use of evidence as either limited to specific topics or as an optional feature that could be implemented only under favourable circumstances. These findings are in line with the evidence available in the literature [20–23]. 

It is not possible to know where the negativity towards implementing EBP stems from. However, it seems likely that it is a combination of undergraduate education, where the new graduates were not fully equipped with the necessary skills to implement EBP knowledge or the dental practice environment and culture they enter into, as part of VDP, which is not conducive to its implementation. The findings from this support assert that they did not feel in a position to deliver care to patients according to available evidence and recommendations for best treatment approaches, even though they had fulfilled their academic requirements and were deemed competent in this regard.

For the education side of this, the problem may lie in the unobserved dimension of the teaching process. This dimension pertains to the behaviour of teaching staff towards EBP, known as the “Hidden Curriculum.*”* [24] The “Hidden Curriculum” suggests that educators inadvertently communicate their values and attitudes, which then trickle down the hierarchical chain of the teaching process. This communication can occur through subtle cues that conveys a certain stance. Research supports the notion that learners are more susceptible to being influenced by this implicit side of teaching when they lack the confidence to develop their own values and attitudes toward certain issues. Consequently, they adopt the attitude of their senior figures. Thus, if students witness educators disregarding research evidence in favour of personal opinions or anecdotal evidence, they are more likely to adopt a similar approach towards EBP. This misalignment in values and attitudes may explain the mixed views on EBP held by dental graduates in this study, despite receiving formal education on the subject during their dental training. While the concept of the hidden curriculum has been extensively investigated in other medical disciplines, [25, 26] there remains a scarcity of evidence related to the dental context, highlighting the need for further investigation. Notably, the influence of this concept may also extend to the attitudes of VDT trainers, adding another layer to the complexities surrounding EBP in dental education and practice.

In addition to the attitudinal barriers, the environment of general dental practice presented significant challenges to the adoption of EBP. The fast-paced nature of dental practice, coupled with perceived financial constraints, seemed to create additional hurdles. The participants highlighted the NHS system as a consistently frustrating element in the decision-making process. The rigidity of the system and its policies negatively impacted the ability of dental graduates to implement what they considered to be best for their patients, creating ethical dilemmas for them. The findings of the study also revealed concerns regarding legal issues, leading to excessive referrals and fostering a culture of “defensive dentistry.” These challenges were not only stressful for the participants of this study but for the GDPs in general practice, as evidenced in previous studies [27, 28]. 

The diary logs provided further insight into how participants adapted their practice to conform to NHS dentistry. For example, Tom expressed increased anxiety about time management and the financial situation. Hence, he adapted his practice to align with the requirements of NHS dentistry. These adaptations are influenced by the guidance and support of their VDT trainers. The participant perceived this support positively as it prepared them for the real-world dental profession.

These views are based on the assumption that EBP is only associated with sophisticated, expensive and time-consuming approaches, which is not necessarily true. Recent evidence supports minimally invasive dentistry and the use of simple, cost-effective strategies to manage dental caries (e.g. non-restorative cavity control) [29–31]. A pilot study was conducted in North Ireland to assess changes in GDPs’ clinical activity when the NHS remuneration system was modified to incentivise prevention treatment planning (along with other positive outcomes) [32]. It found a general reduction in clinical activity including prevention procedures, and although the changes in the payment system were beneficial in various ways, GDPs’ treatment choices quickly returned to the baseline once the incentives were stopped. There are also examples from developed countries where dental care is chiefly offered through private channels and there is still emerging evidence from those countries reporting issues regarding dentists’ behaviour and attitude towards EBP [33–35]. These findings challenge the prevailing notion of the participants views, that more time or better financing would lead to an increase in adoption of EBP.

### Strategies to enhance EBP adoptions

Importance is placed on EBP by the General Dental Council, the Professional Statutory Regulatory Body who oversee dentistry, undergraduate curricula content and delivery by Dental Schools. Our study highlights the complexities of the challenges in EBP adoption among dental graduates, prompting the need actionable strategies for improvement. These strategies could encompass faculty training, which is a critical step in integrating EBP principles into curricula. Training for VDT trainers is equally vital, as they significantly influence the professional identity of new graduates during their early years in practice.

To achieve more ambitious goals, establishing an EBP-friendly environment is vital. This involves ensuring convenient access to evidence-based resources, including online libraries and databases, and user-friendly platforms. Introducing incentives and rewards for actively implementing EBP in clinical practice can serve as a catalyst for widespread adoption [36]. Empowering patients with information is a crucial step to enhance EBP adoption. By fostering a transparent and open dialogue during consultations, dentists can provide clear insights into various treatment options, associated risks, and expected outcomes. Actively encouraging patients to ask questions and involving them in decision-making not only builds trust but also promotes a culture of shared decision-making rooted in evidence [37]. These initiatives necessitate substantial changes in the dental practice landscape, requiring collective efforts amongst regulatory bodies, dental associations, policymakers, and educators to collectively facilitate EPD implementation.

### Study limitations, mitigation strategies, and transferability

While this study provides valuable insights, it is essential to address its limitations and propose strategies to mitigate them. The focus on dental graduates from a single institution during their VDT phase may raise concerns about the generalisability of the findings. Variations in curricula and teaching approaches across dental schools in the UK can also influence dental graduates’ attitudes and behaviours toward EBP. To address these limitations, future research should involve a more diverse sample of dental schools, investigating variations in EBP education and its impact on graduates’ practices.

Another limitation of this study is the relatively short-term follow-up of the new dental graduates on their EBP journey. To address this limitation, future research should consider conducting longitudinal studies that follow graduates over an extended period. Such studies will assess the long-lasting impact of EBP teaching and training on dentists’ routine practice.

Both used of the data collection methods used are qualitative in nature, introducing a degree of subjectivity into the data [34]. We took measures to ensure the quality of analysis, including consulting the literature, expert oversight, and internal coding checks and triangulation with the the quantitative data collected as part of the project [11]. We employed a codebook and reflexive thematic analysis, recognising that the findings are primarily interpreted through the researcher’s lens but remained open to alternative interpretations.

This research is transferable to other contexts as researchers and educators can draw upon the evidence presented here by adapting our strategies and recommendations. For instance, this study highlighted the importance of faculty training in EBP which is likely to be relevant to multiple contexts. extend it to their specific educational settings. This involves equipping educators with the necessary knowledge and skills to intentionally teach and communicate EBP principles. Moreover, training should be extended to immediate educators, such as VDT trainers, who play a critical role in shaping the professional identity of new graduates.

## Conclusion

This study sheds light on the gap between the expected incorporation of EBP values and the actual professional behaviour of new dental graduates. It challenges the belief that undergraduate education alone can guarantee the active use and application of EBP by new dental graduates. Various factors impede the process of EBP implementation. The findings suggest that time and financial constraints within the NHS system, coupled with the views of the trainer and other staff and misconceptions about EBP, serve as barriers to its explicit use in dental practice settings. To address these challenges, further investigation is required to uncover the hidden aspects of dental education and training that contribute to attitudinal obstacles. By gaining a comprehensive understanding of these factors, effective strategies can be developed to cultivate lifelong learning and foster the integration of EBP among dental graduates.

### Electronic supplementary material

Below is the link to the electronic supplementary material.

Supplementary Material 1

## Data Availability

The datasets generated during the current study are available upon reasonable request from the corresponding author.
