# Supplementary material for: New dental graduates transition into UK professional practice; a longitudinal study of changes in perceptions and behaviours through the lens of evidence-based dentistry
Source: BMC Med Educ. 2024 Feb 26;24:195. doi: 10.1186/s12909-024-05182-y (PMC10895742; doi:10.1186/s12909-024-05182-y)
Supplement: Supplementary file 1 — Supplementary Material 1 [file 12909_2024_5182_MOESM1_ESM.docx]

Supplementary materials:

New Dental Graduates Transition into UK Professional Practice; A Longitudinal Study of Changes in Perceptions and Behaviours through the Lens of Evidence-Based Dentistry

# Appendix 1a: Participant information sheet for the semi-structured interview and LADs

University of XXXXX

Title: Changes in new dental graduates’ behaviour and self-efficacy towards guideline compliance in daily practice, during the transition from graduation through Vocational Dental Training

Investigator xxxxxxxx (University of xxxxx).

Phone: XXXXXX

**Invitation to take part in a research study**

You are being asked to take part in a research study. Please read this explanation about the study and its risks and benefits before you decide if you would like to take part. You may take as much time as you need to make your decision. You should ask me to explain anything that you do not understand and make sure that all of your questions have been answered before signing this consent form. Before you make your decision, feel free to talk about this study with the research team you wish. Participation in this study is voluntary.

**What to expect**

The purpose of this study is to explore the transition period from undergraduate studies to independent practice, involving various behaviour constructs of new dentists towards evidence-based practice (EBP). We are also interested in the possible factors that might foster or hinder the use of clinical guidelines in daily practice. There is a lack of research investigating this crucial period of time and how it might influence new dentist’s future clinical decision-making. This is why this research is being conducted.

You have been asked to take part in this research study because you are about to become a new registered dentist, having recently graduated from the University of XXXX. If you choose to participate in this study, you will be invited to complete two interviews with 6-9 months’ time gap. The questions will relate to your experience of transitioning to independent practice and your thoughts/views regarding Evidence-Based Practice. Interviews will be at a location that is convenient to you and should take 60-75 minutes. You will also be asked to participate in doing an audio diary for 6-9 months. You will be requested to talk about your experiences, feelings and thoughts during this important time of your professional practice.

**Time commitment**

Those who complete this consent form will be asked to do an interview for approximately 60-75 minutes. You will not be asked to provide any information that is sensitive in nature or poses contradiction to the confidentiality required by your employer. The interview time and setting will be determined by your preference. The interview will be tape recorded by me. At this time, I will also take notes on your expressed emotions during the interview. An interview guide will be used.

As a separate part of the study, you will also be invited to record regular audio tapings of approximately 6-8 minutes for 6-9 months, and whenever they felt like it (preferred weekly). Audio diaries may be done at any location and may be any length.

**Risks**

The study is considered to be minimal risk.

**Termination of participation**

Your participation in this study is voluntary. You can choose to participate, choose not to participate, and/or withdraw from participation at any time without consequences. Participation will be completely confidential. You may refuse to answer any question you do not want to answer.

**Confidentiality/Anonymity**

The information that is collected for the study will be kept in a locked and secure area by myself at the University of XXXXX for 10 years, after which it will be disposed of as confidential waste.

Any information about you will have a code and will not show your name or address, or any information that directly identifies you. All information collected during this study will be kept confidential and will not be shared with anyone outside the study. You will not be named in any reports, publications, or presentations that may come from this study. If you decide to leave the study, you will be given a choice as to whether you will allow me to use your data or not and your decision will be respected.

Your telephone number and e-mail address will be required for communication purposes to arrange interview sessions. Your preference for form of communication will be respected. This information will be stored separately from the data on an encrypted USB memory stick. All contact details will be destroyed by me at the end of the study period if not previously destroyed due to personal request.

**For further information about this research study**

If you have any questions, concerns or would like to contact me for any reason, please e-mail me at XXXXXX

Everything that you discuss will be kept confidential

# Appendix 1b: Consent form for the semi-structured interview and LADs

University of XXXXX

| *Please tick the appropriate boxes* | Yes |
| --- | --- |
| Taking Part |  |
| I have read and understood the project information sheet dated DD/MM/2018. | o |
| I have been given the opportunity to ask questions about the project. | o |
| I agree to take part in the project. | o |
| I understand that my taking part is voluntary; I can withdraw from the study at any time, and I do not have to give any reasons for why I no longer want to take part. | o |
| I understand that my words may be quoted in publications, reports, web pages, and other research outputs*.* | o |
| Use of the information I provide beyond this project |  |
| I agree for the data I provide to be archived at the University of XXXX/ Dental School | o |
| I understand that other researchers will have access to this data only if they agree to preserve the confidentiality of the information as requested in this form. | o |
| I understand that other genuine researchers may use my words in publications, reports, web pages, and other research outputs, only if they agree to preserve the confidentiality of the information as requested in this form. | o |

Title: Changes in new dental graduates’ behaviour towards guideline compliance in daily practice, during the transition from graduation through Vocational Dental Training

*This is to consent my participate in:*

*Initial and exit interview* o

*Longitudinal audio diaries* o

______________________ _____________________ ________

Name of participant [printed] Signature Date

For further information:

XXXXX

Email: XXXXXX

Thank you for your help

# Appendix 2: Interview guide questions with the NDGs

| Primary questions | 1. Introduction 2. Thank for participation. 3. Questions about the study. 4. Consent. 5. Format of the interview. |
| --- | --- |
| Transition period experience | 1. Describe understanding of transition? 2. Thoughts about the experience so far? 3. Has dental school supported their transition to practice professional? How so? 4. Support expected during your VDT. Expectations around learning during VDT post? VDT trainer 5. *Probe: communication skills, new treatment approach, time management, etc.…* 6. Thoughts about the supports received from various stakeholders (VDT trainer, teaching staff, GDC, NHS, Dental Nurse). |
| Final thoughts | 1. Invite to elaborate on any answers 2. Any additional comments or areas to discuss, that haven’t been covered 3. Thank for time and for sharing thoughts. |

**Appendix 3: Thems and quotes related**

## Upon graduation and before starting professional practice, findings from Interview 1

| Themes | Quotes |
| --- | --- |
| **Theme 1- Expectations:** | “I think that I would quickly go onto the guidelines….before my patient come and just double check what I am doing but I don’t know if I’m being unrealistic”.  P1/Round 1  “I’d make it (my practice) as evidence based as I can, within the scope of what the NHS will allow”.  P2/Round 1  “I would hope so. I would want to check the guidelines to make sure that I was following best practice. I don’t know if I my mentor will encourage me to go online and look it up or they would just tell me what they think I should do”.  P3/Round 1  “So, I don’t really expect go by the book. I think I’ll sort of doing a treatment that would be recognized as common and accepted practice. And then proper consent would protect you”.  P4/Round 1 |

Changes during professional practice, findings from LADs:

| Theme 3- Embarking on professional life: independence and learning curve | *My nurse is also really supportive, she worked with VDPs for about 10 years, she had a really good grasp of the SDR and of treatment codes… She just knew every code, If I said, “What code can I claim for this?” she would say, “1782” or something like that”.*  WK1  I was really nervous when I first started, still sort of shaky, but luckily, you’re still salaried, obviously you still want to get quicker, but it has no outcome on your salary”  WK 1  I was really scared of dealing with real patients who pay for the dental work… I still do, it’s one of the things that Dental School is lacking”  WK3  “I’ve absolutely loved the independence of practicing. I’ve loved being able to make my own treatment planning decisions. I’ve loved being able to carry out procedures without constantly having to get things checked”.  *WK4*  “I’ve learned to place dentine pins for both amalgam and composite restorations. We’ve started doing one file endo technique”.  *WK4* |
| --- | --- |
| Theme 4- Diving into professional practice: Feeling conflicted: | *If you want someone just to be able to say, "Here's what things we can charge a patient." Then you want an accountant. If you want someone that can actually deliver care…then you need to have somebody that knows the science”.*  WK6  I feel the realities of primary care within NHS and independent practice started kicking in…I can’t do gold work or posterior composites when I want because someone has to pay for it…that’s brings a great deal of tension on daily basis”.  WK6  “speed of consultation is becoming a major source of pressure and I would argue probably the greatest area, sort of learning challenge for me is to do consultations rapidly enough, so that I can be an associate Dentist, who is able to actually have a proper income out of my work, as well as being able to deliver decent care for my patients”.  WK6  I’m held more to account if things didn’t go right, it really falls on me and I have to deal with it, it’s not going to be deflected by anyone else, It’s only my name will be at stake here”.  WK8  Some patients are really nice and understanding, but boy some of them are simply just not. I’ve had a couple of patients be difficult about demanding antibiotics, and situations where my temper was tested… trying to remain calm and explain in a professional way why you couldn’t do something”.  WK9  “I had one patient that got pretty angry and yelled at me, which was the first time a patient has ever shouted at me, so that was quite scary, so I tried to stay really calm, I just said to him that his tone was becoming very aggressive and so I would appreciate if he left the surgery”.  WK10 |
| Theme 5- Patient expectations and treatment constraints | I sympathised with the patient because I can quite easily see how that would affect their life, then you're wanting to do as much as you can dentally to help that and to relieve that pressure…but I wasn't able to justify doing any treatment as per the SDR and NHS treatment, although her appearance wasn't optimal”.  *WK11*  “the patient called the practice and accused me of being negligent and having a duty of care towards her, and then asked to be deregistered, that she didn't want to come here for her treatment anymore”.  *WK11*  “I feel really upset about it and actually confused as to why the patient felt that way. I'd put in a lot of effort with that patient to explain to her the reasons for the decisions, that it's not that I didn't want to treat them or that I want them to pay a lot of money privately to have treatment. Just actually, it's a wee bit complicated and requires a specialist's input”.  *WK12*  "If a patient doesn't get what they want, is that just grounds to complain all the time and complain to everyone?... It frightened me a little bit in terms of the claim culture that we have at the moment”  *WK13*  My relationship with my patients, I don’t I think it’s going well (my relationship with my patients)…not really. You probably need to know more stories about why that's the case”.  *WK16*  “I don't necessarily feel that I am looking out for my patients as I should be, as it needs to be. I’m maybe now more anxious than I was”.  *WK18* |

## NDGs behaviour spending six or more months into practice, findings from Interview 2

|  |  |  |
| --- | --- | --- |
| 7a) Ambiguity about the concept: |  | “I read a lot of guidelines myself. So, when you have complicated patients and things and you're like, "Oh. What do I do with this person who I need to take an extraction and they're having blood thinners?" Then I look it up on the SDCEP guideline”.  Charlotte/Round 2  “I would use SDCEP documents. Especially the ‘Guidance in Brief’ ones are quite handy. I always save those to my computer”.  Tom/Round 2  I would say so for most of the time. We’re always trying to get new materials or instruments that have been recommended and try it”.  Carly/Round 2  “No, usually I’ve just gone with clinical use and the word of the Dentists who uses it (new materials)”.  Carly/Round |
|  |  |  |
| 7b) reluctance to use: | “No, I haven’t actually used any of the search tools when it comes to finding evidence in practice”.  Tom/Round 2  “I normally ask my peers. I've got three other Dentists in the practice. I’d ask of them and we just speak about cases".  “You're doing CPD courses, keeping up with what's in now, how to do this crown prep, the best materials. That’s how a lot of people keep up with a lot of things. They wouldn't find that answers themselves, you'd just do a course on it because you have to do CPD anyway”.  Carly/Round 2  John/Round 2 | |
| 7b) reluctance to use: | Attitude | “I'm fairly lazy. I have an impressive stack of BDJs that's about that high that are still pending, which I think goes back to probably 2013 at this point”.  David/Round 2  “Maybe the fact that in General Practice, it's only very rarely that you see something unusual, most of it is sort of bread and butter of Dentistry.”  Carly/Round 2  “I’ve looked up the Perio SDCEP document once. It was like 110 pages and a lot of it was extremely repetitive and I just thought that they could have summarised that a lot better for Dentists because I think it was just way too wordy, this put me off from going back to it again”  P1 /Round 2  “No, definitely no, the others as well. This is not our job, unless we actually were close because we're like, "Let's have a tutorial on appraising articles”.  P3/Round 2  “It was probably something that was more emphasised in undergrad. I don’t think my Trainer really spoke about evidence-based Dentistry very much”.  P9 /Round 2 |
|  |  | “I think that each party is just not paying attention to the others. The GDC wants to make sure that everything is happening the way that protecting the patients. Dental Schools are saying we wanna teach you gold standard treatments. But the second you are going out to practice, we get to deal with the NHS and get to really understand what we can and cannot accomplish, I think they leave it to VDT”.  P3 /Round 2  “I think that as soon as I feel like my skill level is at a good enough standard and that I can stay in an entirely private list. I have no compunction about going over to being entirely a private practitioner. Because then I think I'll be able to provide care that I'm happier with or my patients”.  P1 /Round |
|  |  | “I would say so for most of the time, he does encourage me to try new things, new materials or techniques… I don’t think any of the Trainers do that (encourage VDPs to look for evidence), maybe they are out of that system already. There's this one Trainer in Dunfermline who's involved in all that. He's very interested in research. So maybe then, he might get his trainee to do it. But unless you're with a Trainer that loves research, you probably won't do appraising articles or use PubMed”.  Carly/Round 2 |
|  | NHS | “If you can afford to use the full range of Endodontic files (instead of part of it), you're either in private referral practice for endo, or you're in the hospital environment where you just don't quite have that restriction (money)”.  Emma/Round 2  “We're putting the patient's in the position of saying," you can do, A or B. A is quite basic but available in NHS. B is the one that’s better, it’s evidence-based but it’s private”.  Tom/Round 2  “You have no idea how tedious the environment we're in. Dentists being hemmed between the rock that is funding and the hard place is, "Do you see standards". We already see it where Dentists are, cutting corners in ways to try to make things work in order to square this problem, and then they are following that, and we start to see the Dentists on the other side, ‘Well, either I can't provide NHS treatment’ ”.  Camron/Round 2  “I am their Dentist. I still want to just do a good job and don’t want to change my values by trying to sell private treatments to patients”.  “The NHS is unfairly telling us, "Well you've got to provide the one that's the better one. But we're not gonna pay you anything more for it."  P9 /Round 2  It’s creating a moral hazard by steering people in that direction cause patients will make economic choices about their healthcare, and they'll say, "Well, the best treatment for me would cost £60, but I don't really have £60, so I'm going to have to go make a compromise of a treatment for 10”.  P4 /Round 2  “There's increasing cost of living, rents go up, and utility costs go up. Meanwhile, the, the compensation paid to Dentists for working in the NHS is just not keeping up at all”.  P2 /Round 2  If a treatment plan went over a certain amount of money, then I had to request prior approval before I could continue the patient’s treatment plan. That generally can take quite a long time, and sometimes is quite difficult”.  P3 /Round 2  “Making speed is a way of compensating for the fact that the standards are poor. You either have high standards or high turnover. That is how you make a business work”.  P5/Round 2 |
